# Automated Construction of Node Software Using Attributes in a Ubiquitous Sensor Network Environment

**DOI:** 10.3390/s100908663

**Published:** 2010-09-17

**Authors:** Woojin Lee, Juil Kim, JangMook Kang

**Affiliations:** 1 Department of Information and Communication Engineering, Sejong University 98 Gunja-Dong, Gwangjin-Gu, Seoul 143-747, Korea; E-Mail: woojin@sejong.ac.kr; 2 R&D Center, Hunter Technology, 170-5 Guro-3Dong, Guro-Gu, Seoul 152-769, Korea; E-Mail: sespop@empal.com; 3 Electronic Commerce Research Institute, Dongguk University, 707 Seokjang-dong, Gyeongju, Gyeongsangbuk-do, 780-714, Korea

**Keywords:** attributes, node software, ubiquitous sensor network application, automated construction, model-based development

## Abstract

In sensor networks, nodes must often operate in a demanding environment facing restrictions such as restricted computing resources, unreliable wireless communication and power shortages. Such factors make the development of ubiquitous sensor network (USN) applications challenging. To help developers construct a large amount of node software for sensor network applications easily and rapidly, this paper proposes an approach to the automated construction of node software for USN applications using attributes. In the proposed technique, application construction proceeds by first developing a model for the sensor network and then designing node software by setting the values of the predefined attributes. After that, the sensor network model and the design of node software are verified. The final source codes of the node software are automatically generated from the sensor network model. We illustrate the efficiency of the proposed technique by using a gas/light monitoring application through a case study of a Gas and Light Monitoring System based on the Nano-Qplus operating system. We evaluate the technique using a quantitative metric—the memory size of execution code for node software. Using the proposed approach, developers are able to easily construct sensor network applications and rapidly generate a large number of node softwares at a time in a ubiquitous sensor network environment.

## 1. Introduction

Recent advances in wireless communications and electronics have enabled the development of low-cost, low-power, multi-functional sensor nodes. These sensor nodes leverage the idea of sensor networks [1]. A Ubiquitous Sensor Network [2] is a wireless network which consists of a large number of lightweight, low-powered sensor nodes. Such sensor nodes consist of sensing, data processing and communicating components. Sensor networks are drawing a lot of attention as a way of realizing a ubiquitous society. They collect environmental information to realize a variety of functions through a lot of wireless nodes that are located everywhere [1,3]. The nodes are connected to a network and sense geographical and environmental changes of the field. Objects can recognize other objects through the sensor network and perceive changes in the environment. Users can obtain and use information at any time in any place through the objects connected to the sensor network. The sensor networks can be used for various application areas such as health, military, robot, home and so on.

However, the construction of applications is challenging. Node resources in a sensor network are limited and wireless communication between nodes is unreliable. Nodes should also perform low-power operations. Accordingly, techniques to help developers easily construct applications, even if they do not know the low-level information details such as low-level communication, data sharing and collective operations, are necessary. Moreover, a sensor network consists of a large number of sensor nodes that have various roles and a large number of node softwares for those roles must be constructed, so such techniques should also help automatic construction of applications in order to efficiently generate a large amount of node software.

To satisfy these requirements, this paper proposes a technique for automated construction of node software using attributes. In the proposed technique, application construction proceeds by first developing a model for the sensor network. Then design of software for nodes in the sensor network is achieved by setting the values of the predefined attributes. After that, the sensor network model and the design of node software are verified. Finally, source codes of node software are automatically generated from the sensor network model.

In this paper, we describe in Section 2the limits of the existing techniques for USN application development by comparing them with our approach. In Section 3 we present the USN application development framework which is the base for the proposed technique. We describe the process and method for constructing an application based on the proposed framework in Section 4. To make our illustration more concrete, we describe the proposed technique using a gas/light monitoring application as an example. In Section 5, we evaluate the proposed technique through a case study with a Gas and Light Monitoring System based on the Nano-Qplus operating system. We evaluate the proposed technique using a quantitative metric—the memory size of execution code for node software. Section 6 concludes the paper. The contribution of this paper is to demonstrate that the proposed technique helps developers easily construct sensor network applications, generates a large number of node softwares at a time and provides methods to verify a sensor network model.

## 2. Related Works

There are currently several techniques for generating USN applications such as Regiment [4], Kairos [5], SNACK [6], SPIDEY [7], TinyGALS [8] and ATaG [9–11]. SNACK and TinyGALS draw up the model at the node-level and develop the node software, while Regiment, Kairos, SPIDEY and ATaG draw up the model at the network-level and develop an application. Techniques at the node-level provide methods of developing single software to be implemented at each node. On the other hand, network-level techniques provide methods of designing the model with focus on actions among multiple nodes at the network-level and, on that basis, developing node software to be implemented at each node. Therefore, because the network-level method generates node software for multiple nodes comprising the sensor network on the basis of the single sensor network model, it is more effective than the method at the node-level that individually designs and generates node software for each node. Accordingly, this paper will present a method of drawing up the model and developing an application at the network-level in order to develop sensor network application more effectively.

TinyGALS generates software codes from the model. Therefore, the model should be designed in detail to include sufficient information to generate software codes. Regiment, Kairos, SNACK, SPIDEY and ATaG provide high level languages or scripts for application design. If using these techniques, the programs should be prepared by using the provided languages in order to generate an application. On the other hand, the method presented in this paper designs an application by setting values for the pre-defined attributes so that it can design the application more conveniently than other methods.

The attributes which are mentioned in ATaG [9–11] are similar to our attributes in terms that the attributes represent capability of a node, but they are used to determine the type of a node. In our approach, attributes represent functional capability of the node, so developers can program node software using the attributes. In contrast with our approach, developers must program tasks using the provided langauge for implementing functional capability of the node in ATaG [9–11].

SNACK conducts model validation by finding errors (for example, unknown component types, multiple declarations of the same name, missing parameters, connections that join interfaces with different types and so forth) through static semantic checking. TinyGALS conducts model validation by finding errors through syntax checking. On the other hand, ATaG conducts model validation in two stages during compilation. First, it checks whether task/data names are duplicated, whether one data item has two producers, *etc.* through a syntax check. Second, it makes sure that application codes will be generated as intended by the developer by conducting task mapping according to instantiation rules. Such model validation is to confirm that the application codes will be generated as requested by the developer. In this paper, validation will be conducted by finding errors through syntax check during compilation and further, for more accurate model designing, model verification will be conducted before compilation to find errors that are likely to occur in the model design process. As model design is conducted by a developer, errors can occur in the design process due to mistakes and faults of the developer. In order to check such errors beforehand, this paper conducts model verification at the stage prior to generation of application codes from a model.

There are also modeling tools that achieve easy and rapid programming such as LabVIEW [12] and RTDS [13]. However, they are the tools for embedded application development and are not suitable for sensor network application development. They support embedded operating systems but embedded operating systems have different characteristics from sensor network operating systems, which are lightweight and low-power and capable of controlling resource-constrained hardware platform, so such tools cannot be easily adopted for developing applications for sensor network operating systems. Moreover, when using these tools node softwares can only be developed one at a time. This contrasts with our approach, which allows a large number of node softwares to be generated at once from the same model.

## 3. USN Application Development Framework

**Figure 1.**
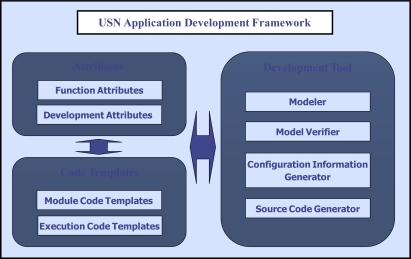
USN application development framework.

When our approach is used, node software for an application is designed by setting values of the predefined attributes and then source code for node software is automatically generated by composing code templates according to the values of these attributes. To support this application development technique, the USN application development framework should be established. Figure 1 presents the framework.

The USN application development framework consists of Attributes, Code Templates and Development Tool. To effectively develop USN applications using the method presented in this paper, a toolkit for application development needs to be established. We call this toolkit “Development Tool.” The Development Tool is established by an expert developer familiar with the target OS for which USN application will be executed.

The developer of the Development Tool makes Attributes and Code Template necessary for designing an application on the basis of functions and modules (or components) provided by the target OS. The Attributes and Code Templates so made will be included in the Development Tool and used in developing USN applications.

Attributes are categorized into Function Attributes and Development Attributes. Function Attributes are used to select the modules which are provided by the target OS. Development Attributes are used to set the information for development of node software.

Code templates are developed for generating node software for an application according to the functions of nodes such as data sensing, data transmitting, data collecting, data processing and actuating. Code templates are developed based on modules provided by target OS. Code Templates are categorized into Module Code Templates and Execution Code Templates. Module Code Templates are developed by composing the modules of target OS in order to support the functions of nodes. Execution Code Templates are templates for core codes to execute node software based on the selected functions. Code Templates can vary according to the target OS.

Finally, the role of the Development Tool is to support sensor network programming in order to expedite development of applications. The Development Tool includes core four modules—Modeler, Model Verifier, Configuration Information Generator and Source Code Generator. Modeler helps developers to draw sensor network model diagrams and design node software by setting attributes. Model Verifier checks whether the model of the application is correctly designed without errors. The Configuration Information Generator creates the configuration information of nodes in the model using the model information such as attribute values. The Source Code Generator creates software for nodes using code templates.

## 4. USN Application Development Based on the Framework

### 4.1. The Process of USN Application Development

**Figure 2.**
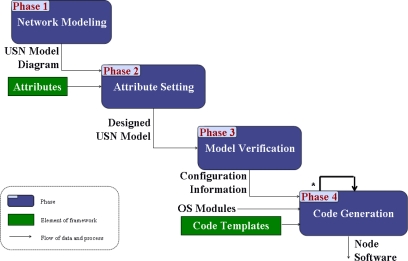
Construction process of USN application.

Figure 2 shows the process for constructing a USN application based on the framework proposed in our approach.

In Phase 1 of the process, the developer draws a sensor network model diagram. The model is described using the UML class diagram notation [17]. In Phase 2, the developer sets attribute values of classes in the model. Through the setting of attribute values, operating system components to support the application are selected. In Phase 3, the developer verifies the sensor network model. If the model is not correct, the construction process should be repeated from Phase 1 or Phase 2. Model verification of Phase 3 is necessary because the developer manually writes the model and sets attribute values. After model verification, the developer generates configuration information for each class in the model. In Phase 4, the developer generates source code for classes using the configuration information and code templates. Then application development is completed by editing the source code if needed.

### 4.2. Sensor Network Modeling

A sensor network model is described with three elements: node, node type and association. The notation of the sensor network model shown in Table 1 was obtained from UML class diagram notation [17].

There are four roles which can be performed by nodes in a sensor network: sensor, router, sink and actuator. In sensor network modeling phase, a developer should draw a sensor network model diagram by considering the features of nodes according to their roles. Features per node role are as follows.

- Sensor: A node which has a sensor role senses data and transmits the data to a coordinator node.
- Router: A node which has a router role plays the coordinator role. It controls a subnetwork. A router node receives data from other nodes in the subnetwork and transmits the received data to the Personal Area Network (PAN) coordinator node.
- Sink: A node which has a sink role plays the PAN coordinator role. It controls the whole network. A sink node collects data from other nodes in the sensor network and controls them.
- Actuator: A node which has an actuator role controls devices.

Node Type can be created according to the roles for nodes. The “type name” is defined by enumerating role names separated by underscore. For example, type of a node is <<SENSOR>> if the node has a sensor role. And type of a node is <<SENSOR_ROUTER>> if the node has two roles—sensor and router.

### 4.3. Attribute Setting

**Figure 3.**
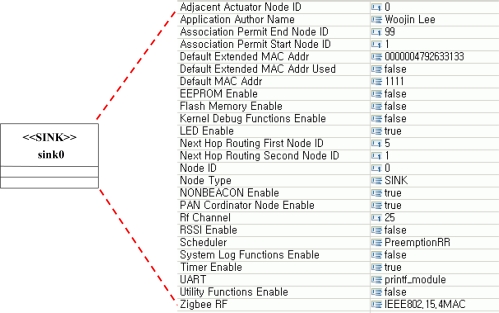
Attributes values setting for sink0.

To generate node software from the designed model, the developer should configure the attributes of each node. The developer should set attribute values of each class in the model. The developer can set scheduler type, network topology, sensor type, *etc.* of each class in the model. Figure 3 shows an example of attribute setting. In the example, one can see attribute values of sink0 in the sensor network model for Gas and Light Monitoring System. A developer can design node software by setting attribute values for each node in the sensor network model as in Figure 3.

### 4.4. Model Verification

Model verification is conducted to find and correct errors of the designed model. The developer conduct model verification to check whether the model he designed is accurately designed according to specifications and assumptions [18]. If the model has errors, the generated application is highly likely to produce errors during execution. Therefore, model verification is needed to check before generation of application codes whether there are errors in the designed model.

For this, this paper conduct model verification on the designed sensor network model from the three viewpoints of commonality verification, association verification and node verification.

- Commonality verification: Requirements common to all nodes should be confirmed in order to ensure that communication between nodes can be performed without any problems. They include communication protocol compatibility and communication channel compatibility.
- Association verification: Associations in the sensor network model represent the routing paths between nodes. Through association verification, developers can check whether the model is properly designed such that data is transmitted to the server through an appropriate routing path. Association verification is effective in case of designing an application using a static routing table. A static routing table may be used when the number of nodes comprising the sensor network is small like in a home network and the application to be designed can determine the routing path between the nodes beforehand. A static routing table may also be used when designing an application based on an OS that supports static routing such as Nano-Qplus. In such cases, conducting association verification is effective in generating an accurate application. If the operating system supports dynamic routing, association verification of the sensor network model is not performed.
- Node verification: Attribute values of each node should be checked for their correct values. Through node verification, developers can check whether node software is properly designed such that they satisfy the constraints imposed by the node type and the constraints imposed by the target platform.

After model verification, the developer should generate configuration information for each node before code generation, which is automatically generated from model information. The configuration information stores attribute values which are set in Phase 2. And it is used to automatically generate node software for each node.

### 4.5. Code Generation

**Figure 4.**
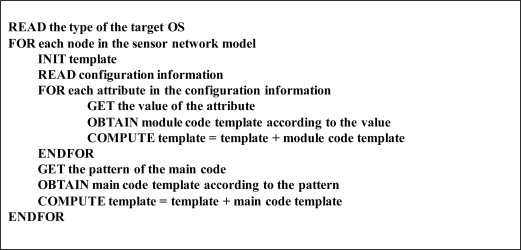
The algorithm for generating node software.

Figure 4 presents the algorithm for generating software source codes of each node. Configuration information is parsed in order to get the attribute values of a node and to select the operating system components that are necessary to generate the software. According to the values of attributes, module and execution code templates are selected from the Code Templates Repository and composed into the node software.

In general, even when two nodes have the same node type, their software source codes may differ from node to node because different components can be selected depending on the specific values of their attributes.

## 5. Evaluation

We performed a case study in order to confirm the effectiveness of the proposed approach. In this section, we apply the proposed technique to Gas and Light Monitoring System (G&LM System) based on the Nano-Qplus operating system for home environmental monitoring and evaluated the result. A variety of applications using gas sensor networks are developed for environmental and safety monitoring [19,20]. A light sensing and actuation application [21] is a well-studied problem in the home environment. Accordingly, we prototyped G&LM System for home environmental monitoring as an application example.

**Figure 5.**
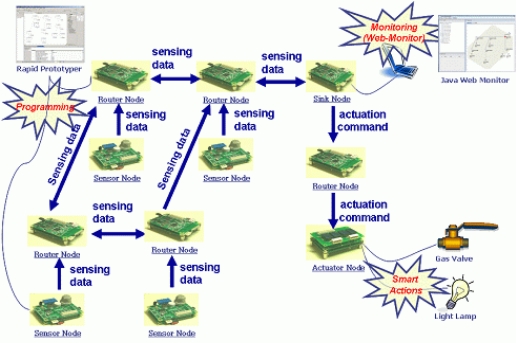
Structure of the G&LM System.

Our sample application consists of nodes which have roles such as gas and light sensor, router, sink and actuator. Sensor nodes sense gas and light data and transmit the sensing data to router nodes. The router nodes receive the data and transmit it to the sink node. The sink node aggregates the data, computes it, determines the action commands and transmits the commands to actuator nodes. The actuator nodes perform the actions, that is, the gas valve and the light lamp operate according to the actions. Figure 5 presents the structure of the G&LM System. The G&LM System is a simple environmental monitoring system, but it has all kinds of sensor nodes which are necessary for a sensor network. So, we think that it is possible to evaluate the proposed technique through the G&LM System.

In this paper, development framework is established first in order to develop an application for G&LM system based on the Nano-Oplus OS. The development framework is established in the order of attribute design, preparation of code templates and development of Development Tool.

Table 2 is the complete list of attributes made for model design of an application based on the Nano-Qplus OS. When the values for the attributes in Table 2 are set, the code templates related to the relevant attributes are selected and combined to generate node software.

Table 3 maps the OS module code files corresponding to each attribute in order to prepare module code templates necessary to generate node software to be implemented under the Nano-Oplus OS.

Table 4 shows module code templates and execution code templates drawn up for generation of node software codes to be implemented under Nano-Oplus OS. Table 5 shows design of the table on determination of reliance between templates necessary for combination of code templates for generation of node software based on Nano-Oplus OS.

Node software is generated by combining code templates in the order presented in Table 5 according to the role of node. The software code is generated based on the templates presented in Table 4. The templates of Table 4 are generated from the module code files presented in Table 3. Using the attributes presented in Table 2, module code files are selected and development information is set to generate node software.

**Figure 6.**
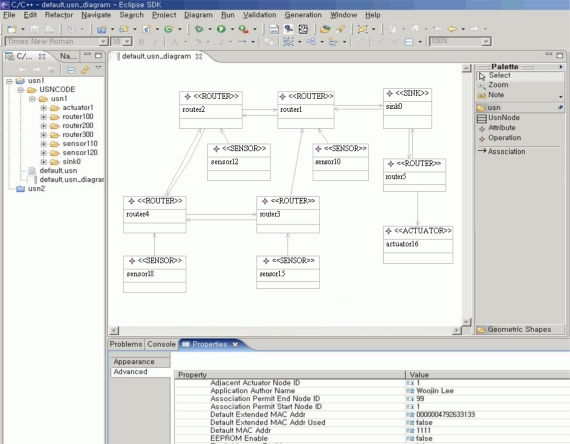
G&LM System modeling using the development tool.

Figure 6 shows the result of G&LM System modeling using the tool to support application development based on the Nano-Qplus operating system. It was implemented as a plug-in for an Eclipse [15] platform and utilized the Eclipse Graphical Modeling Framework (GMF) [16] for the modeling of the application. Through the tool, developers can perform modeling, design and code generation of the application for the Nano-Qplus [14] operating system.

**Figure 7.**
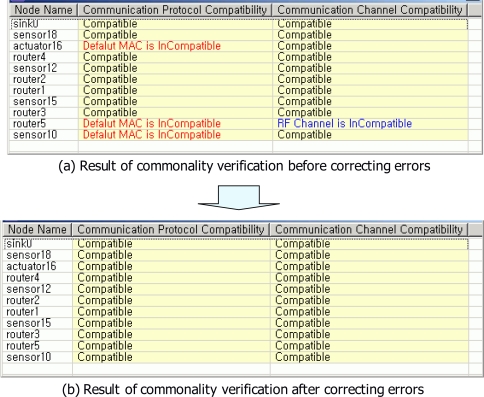
The result of commonality verification.

Figure 7 shows the result of commonality verification. Through the commonality verification, errors for communication protocol and communication channel of a USN model were found. Figure 7(a) shows four errors which were detected in commonality verification. Communication protocols of actuator16, router5 and sensor10 nodes were not compatible because default MAC addresses of actuator16, router5 and sensor10 nodes were set to wrong value. Communication channel of router 5 node was also not compatible because the RF channel of router 5 node was set to a wrong value. Accordingly, default MAC addresses of actuator16, router5 and sensor10 nodes were set to proper values. RF channel of router 5 node also was set to proper value in order to generate correct application. After correcting wrong values, communication protocols and communication channels of nodes were compatible [see Figure 7(b)].

**Figure 8.**
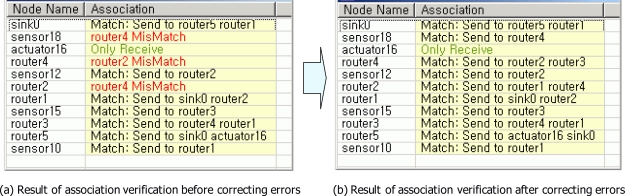
The result of association verification.

Figure 8 shows the result of association verification. Through the association verification, errors for associations between nodes in a USN model were found. Figure 8(a) shows two errors which were detected in the association verification. Figure 8(a) presents the mismatched associations. The association between sensor18 node and router4 node was mismatched. The association between router4 node and router2 node was also mismatched. Accordingly, the mismatched associations were corrected. After correcting the mismatched associations, there were no errors in associations between nodes [See Figure 8(b)]. That means routing path of the sensor network for light & gas monitoring application is correct.

**Figure 9.**
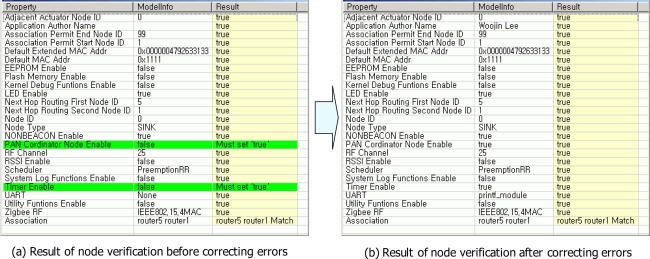
The result of node verification for sink0.

Figure 9 shows the result of node verification for sink0 node. Through the node verification, errors for each node in a USN model were found. Figure 9(a) shows two errors which were detected in node verification for sink0 node. Sink0 node should be PAN coordinator and it should use timer module. Accordingly, values of PAN_Cordinator_Node_Enable and Timer_Enable were set to ‘true’ in order to generate correct software codes for sink0 node. After correcting wrong values, all attribute values of sink0 node were appropriate [see Figure 9(b)].

**Figure 10.**
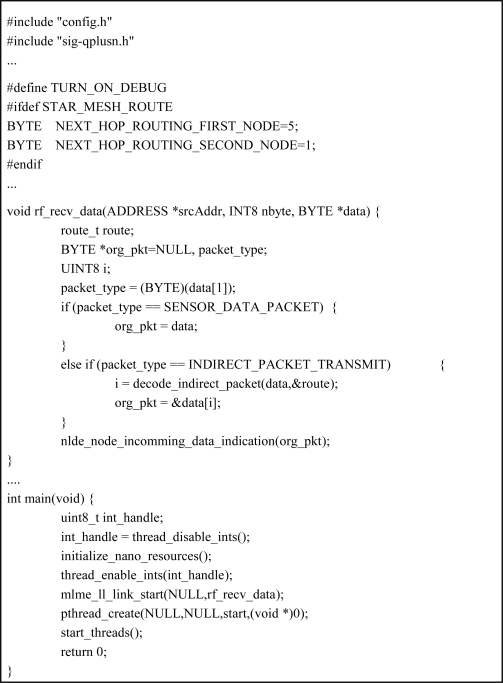
Generated source code for sink0.

Figure 10 shows the automatically generated software codes for sink0 node. Sink0 node receives values of gas and light sensed by the sensor node and compares them with threshold values. If it determines on comparison that gas valve or light lamp needs to be operated, it transmits through router node actuation command to actuator node connected to gas valve or light lamp. Figure 10 is part of software codes implementing such operation, which shows module part that processes transmitted data and execution code part.

In order to evaluate the efficacy of the proposed development approach, we developed the application for G&LM System using two approaches and compared the results from the two approaches. One approach is to develop the application using the proposed technique as you can see in the previous sections. Another approach is to manually develop the application using a standard set of APIs which are provided by the target operating system. We developed the application for G&LM System based on the Nano-Qplus operating system version 1.5.x in two approaches. The evaluation environment is as follows:

- Developer—Expert (Known about Nano-Qplus & USN concept)
- System—AMD Athlon XP 2600(CPU), 1Gbytes(RAM), Windows 2000
- Nano-Qplus Version—1.5.2e

We compared the memory size of execution code in the two cases. Table 6 presents the result of comparison. The first one is the size of node software generated when toolkit is made by the presented method and the application is established by using the toolkit. The second one is the size of node software when the developer directly developed the application using APIs provided by the sensor network OS without using the presented method. From the results presented in Table 6, we find that the memory size of execution code is approximately the same in the two cases. That is, the execution code produced by our approach is optimized. In our approach, the attributes are used to compose modules or components of target operating system, so the execution code sizes are not increased.

## 6. Conclusions

The traditional techniques for generating sensor network applications are limited in that developers must learn new abstraction mechanisms such as high-level language and APIs and most of the approaches do not provide methods to verify models of applications. Moreover, a developer can generate only one node software at a time in some approaches. To complement the existing techniques, this paper proposed a programming technique that helps users construct a large number of node softwares for sensor network applications easily and rapidly.

The suggestions in this paper can be divided into two—one that constitutes framework and the other that develops the sensor network application. In the framework build, the method to construct development kit on an attribute basis is proposed. The framework is constructed by professionals who are well aware of the sensor network operating system. The framework provides the infrastructure so that node software for sensor network application can be created automatically as in SNACK [6]. The codes of sensor network operating system are modularized in the framework. Therefore, a new template does not have to be added nor an existing template need be altered unless the operating system codes are updated. This framework can be used continuously once constructed by the professionals who know sensor network operating system well, unless the operating system is changed for a new one.

In the section of sensor network application development, the method to develop node software for sensor network application through attribute set-up is proposed so developers who do not know the sensor network operating system can create applications easily. Developers who do not know operating system code can easily develop sensor network applications because the node software is created automatically through attribute set-up offered by framework. In other words, sensor network applications can be developed much easier than with other existing techniques, as node software is created only by attribute set-up on the basis of framework constructed by specialists, not by each developer. This way is different from the existing techniques in which each developer must make codes for the each node software. Of course, existing code is reusable in the techniques but if a node with new function is added, a new code should be made accordingly. So, developers should be familiar with code writing. However, if developers follow what we suggest in this paper, they just need to set up the attribute value for the relevant node even if new nodes are added.

The development using attributes is the key technique that enables automated construction of node software for USN application. The proposed technique has the following strengths:

- Using attributes, the developer can easily design applications without learning new abstraction mechanisms.
- Since the technique generates code from sensor network model instead of models of node software, a large number of node softwares can be generated at once. For example, if a developer designs a model for a sensor network which consists of 50 nodes, node software for the 50 nodes can be generated from the model at one time. Node software here refers to an image file in binary format that can be installed in one node and can be composed of multiple source code files.
- The technique provides a method to verify the sensor network model so that developers can verify models of applications in terms of commonality verification, association verification and node verification.

Table 7 shows the result of comparison and evaluation between the method presented in this paper and the existing USN application development methods mentioned in relevant studies. As seen in the Table, the method presented in this paper has the advantages of design facilitation and generation of more accurate applications as well as all the advantages of the existing methods. As future research directions, we plan to extend the scope of the verification aspects that can be checked by our verification module and also plan to build Model Simulator for strengthened verification and validation of sensor network models based on formal approaches.

## Figures and Tables

**Table 1.** The notation for the sensor network model.

**Name**	**Notation**	**Corresponding UML notation**
Node	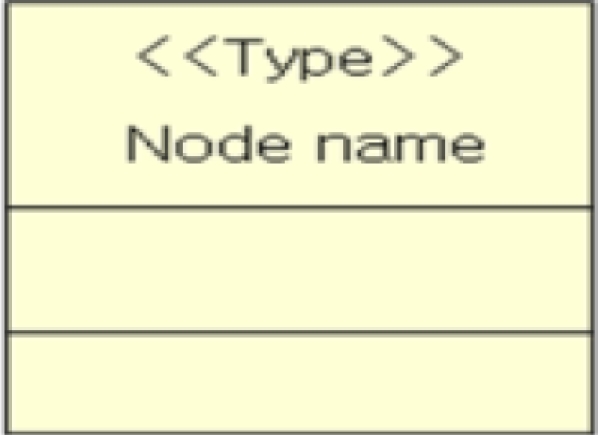	Class
Node Type	<<type name>>	Stereotype which represents the type of a class. <<SENSOR>>, <<ROUTER>>, <<SINK>>, <<ACTUATOR>>, <<SENSOR_ROUTER>>, <<ROUTER_SINK>>, <<ACTUATOR_ROUTER>>, *etc.* can be stereotypes of the classes that indicate node types
Association	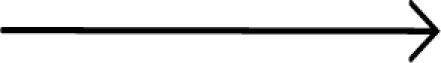	Association between classes

**Table 2.** The list of attribues for the design of node software based on Nano-Qplus.

**Attribute**		**Description**

Development Attributes	nodeType	Choose one type among Sensor, Router, Sink and Actuator.
	applicationAuthorName	Write information about application author.
	nodeID	Write identification number of node.
	adjacentActuatorNodeID	Write identification number of adjacent actuator node.
	PAN_Cordinator_Node_Enable	Decide whether the node is PAN coordinator.
	NON_BEACON_Enable	Decide whether the node uses BEACON.
	defaultMACAddr	Write MAC address.
	Default_Extended_MAC_Addr_Used	Decide whether the node uses extended MAC address.

	defaultExtendedMACAddr	Write extended MAC address if the node uses extended MAC address.
	associationPermitStartNodeID	Write identification number of the first node which is permitted to associate.
	associationPermitEndNodeID	Write identification number of the last node which is permitted to associate.
	nextHopRoutingFirstNodeID	Write identification of the next node in routing path.
	nextHopRoutingSecondNodeID	Write identification of the next alternative node in routing path.
	rfChannel	Write RF channel. If nodeType is Router or Sink, rfChannel should be set.
	scanChannel	Write scan channel. If nodeType is Sensor or Actuator, scan Channel should be set.

	Scheduler	Choose one among none, FIFO and PreemptionRR.
Function Attributes	Zigbee RF	Choose one among Simple, IEEE802.15.4MAC and StarMesh.
	EEPROM_Enable	Enable EEPROM module.
	Flash_Memory_Enable	Enable Flash Memory module.
	Timer_Enable	Enable timer module.
	UART	Choose one among none, printf_module, scanf_module and printf&scanf.
	LED_Enable	Enable LED module.
	RSSI_Enable	Enable RSSI module.
	Sensor_Battery_Enable	Enable Battery sensor module.
	Sensor_Temperature_Enable	Enable Temperature sensor module.
	Sensor_Light_Enable	Enable Light sensor module.
	Sensor_Gas_Enable	Enable Gas sensor module.
	Sensor_Point_infra_red_Enable	Enable Point_infra_red sensor module.
	Sensor_Humidity_Enable	Enable Humidity sensor module.
	Sensor_Ultra_sonic_Enable	Enable Ultra_sonic sensor module.
	Utility_Functions_Enable	Enable utility functions.
	Kernel_Debug_Functions_Enable	Enable kernel debug functions.
	System_Log_Functions_Enable	Enable system log functions.

**Table 3.** The list of module code provided by Nano-Qplus corresponding to attributes.

**Attribute**	**Module Code Files of Nano-Qplus**

Timer_Enable	timer.h, timer.c
ADC_Enable	adc.h, adc.c
UART	printf.h, printf.c, scanf.h, scanf.c
EEPROM_Enable	eeprom.h, eeprom.c
LED_Enable	led.h, led.c
Scheduler	fifo.h, fifo.c, preemption-rr.h, preemption-rr.c
Zigbee_RF	rf.h, rf.c, net.h, mac.c, routing-star-mesh.c
Flash_Memory_Enable	flashmem.h, flashmem.c
Sensor_Battery_Enable	adc_bat.h, adc_bat.c
Sensor_Temperature_Enable	adc_temp.h, adc_temp.c
Sensor_Light_Enable	adc_light.h, adc_light.c
Sensor_Gas_Enable	adc_gas.h, adc_gas.c
Sensor_Point_infra_red_Enable	adc_ir.h, adc_ir.c
Sensor_Humidity_Enable	adc_humidity.h, adc_humidity.c
Sensor_Ultra_sonic_Enable	adc_ultrasonic.h, adc_ultrasonic.c
Utility_Functions_Enable	utils.h, utils.c
System_Log_Functions_Enable	log.h, log.c

**Table 4.** Code templates for node software based on Nano-Qplus.

**Template Type**			**Template**
Module Code Template	Module code templates for attributes without option		#include “.h file name of a module” #ifdef module name #include “.c file name of a module” #endif
	Module code templates for attributes with option		#include “.h file name of a module” #ifdef module name #if defined.(name of option) #include “.c file name of option” #elif defined (name of option) #include “.c file name of option” #endif … #endif
Execution Code Templates	Codes included according to selection of the type of RF module	SimpleZigbee	mlme_start_request(MY_MAC_ADDRESS, rf_recv_data);
		MAC Zigbee	mlme_ll_link_start(NULL, rf_recv_data);
		StarMesh	
	Codes included according to selection of the type of scheduler	FIFO	(*start)((void *)0);
		PreemptionRR	uint8_t int_handle; int_handle = thread_disable_int(); thread_enable_ints(int_handle); pthread_create(NULL, rf_recv_data); start_threads();

**Table 5.** Table on determination of reliance between templates according to the role of node.

**Role of Node**	**Reliance between Code Templates**

SENSOR	start → net_schedule→sense→ send
ROUTER	start → net_schedule →receive→ send
ACTUATOR	start → net_schedule →receive→ actuate
SINK	start → net_schedule →receive→compute→ send

**Table 6.** The memory size of execution code.

**Node Type**	**The proposed approach**	**Manual development approach**

Sensor	102Kbytes	100Kbytes
Router	100Kbytes	99Kbytes
Sink	103Kbytes	101Kbytes
Actuator	101Kbytes	100Kbytes

**Table 7.** Comparison of the techniques for USN application development.

	**Our approach**	**Regiment**	**Kairos**	**SNACK**	**SPIDEY**	**TinyGALS**	**ATaG**

Programming level	Network-level	Network-level	Network-level	Node-level	Network-level	Node-level	Network-level
Design method	AVS	WP	WP	WP	WP	DM	WP
Model verification	Support	Not Support	Not Support	Not Support	Not Support	Not Support	Not Support
Model validation	Support	Uncertain	Uncertain	Support	Uncertain	Support	Support
Code generation method	Auto	Auto	Auto	Auto	Auto	Auto	Auto
No. of node software generated from model	multiple	multiple	multiple	multiple	multiple	single	multiple
Convenience of design	H	M	M	M	M	MH	M
Accuracy of application	MH	M	M	M	M	M	M

* WP: write program using high-level language or script; DM: detailed model design; AVS: attribute value setting; Auto: automatically; M: medium; MH: medium-high; H: high.
